# An ant–plant by-product mutualism is robust to selective logging of rain forest and conversion to oil palm plantation

**DOI:** 10.1007/s00442-014-3208-z

**Published:** 2015-01-10

**Authors:** Tom M. Fayle, David P. Edwards, William A. Foster, Kalsum M. Yusah, Edgar C. Turner

**Affiliations:** 1 Faculty of Science, University of South Bohemia, Branišovská 31, 370 05 České Budějovice, Czech Republic; 2 Institute of Entomology, Biology Centre of the Academy of Sciences Czech Republic, Branišovská 31, 370 05 České Budějovice, Czech Republic; 3 Forest Ecology and Conservation Group, Imperial College London, Silwood Park Campus, Buckhurst Road, Ascot, Berkshire SL5 7PY UK; 4 Department of Animal and Plant Sciences, University of Sheffield, Western Bank, Sheffield, S10 2TN UK; 5 School of Marine and Tropical Biology, Centre for Tropical Environmental and Sustainability Science (TESS), James Cook University, Cairns, QLD Australia; 6 Insect Ecology Group, University Museum of Zoology Cambridge, Downing Street, Cambridge, CB2 3EJ UK; 7 Institute for Tropical Biology and Conservation, Universiti Malaysia Sabah, Jalan UMS, 88400 Kota Kinabalu, Sabah Malaysia

**Keywords:** Bird’s nest fern, Formicidae, Malaysian Borneo, Oil palm, Rain forest

## Abstract

Anthropogenic disturbance and the spread of non-native species disrupt natural communities, but also create novel interactions between species. By-product mutualisms, in which benefits accrue as side effects of partner behaviour or morphology, are often non-specific and hence may persist in novel ecosystems. We tested this hypothesis for a two-way by-product mutualism between epiphytic ferns and their ant inhabitants in the Bornean rain forest, in which ants gain housing in root-masses while ferns gain protection from herbivores. Specifically, we assessed how the specificity (overlap between fern and ground-dwelling ants) and the benefits of this interaction are altered by selective logging and conversion to an oil palm plantation habitat. We found that despite the high turnover of ant species, ant protection against herbivores persisted in modified habitats. However, in ferns growing in the oil palm plantation, ant occupancy, abundance and species richness declined, potentially due to the harsher microclimate. The specificity of the fern–ant interactions was also lower in the oil palm plantation habitat than in the forest habitats. We found no correlations between colony size and fern size in modified habitats, and hence no evidence for partner fidelity feedbacks, in which ants are incentivised to protect fern hosts. Per species, non-native ant species in the oil palm plantation habitat (18 % of occurrences) were as important as native ones in terms of fern protection and contributed to an increase in ant abundance and species richness with fern size. We conclude that this by-product mutualism persists in logged forest and oil palm plantation habitats, with no detectable shift in partner benefits. Such persistence of generalist interactions in novel ecosystems may be important for driving ecosystem functioning.

**Electronic supplementary material:**

The online version of this article (doi:10.1007/s00442-014-3208-z) contains supplementary material, which is available to authorized users.

## Introduction

When humans severely alter the environment, ecologically stable, novel ecosystems can be created (Hobbs et al. 2009). These ecosystems are characterised by the extinction of some species (Tilman et al. 2001), invasion by others (Mack et al. 2000), and shifts in the abiotic environment (Ewers and Banks-Leite 2013). As a consequence, the network of interactions between species can be significantly altered (Tylianakis et al. 2010), with the loss of individual species leading either to co-extinctions of species reliant on each other (Koh et al. 2004) or increases in abundance of species whose competitors or natural enemies disappear (Ritchie and Johnson 2009). These processes are expected to change the adaptive landscape in newly formed ecosystems, with shifts in the costs and benefits of associations between specific partner species (Aslan et al. 2013).

Mutualistic interactions between species are widespread in both natural and novel ecosystems. Some mutualisms are highly specific, with each partner having evolved to invest reciprocally in the other’s fitness (Foster and Wenseleers 2006). However, it is now clear that generalist mutualisms, including those defined as “by-product mutualisms” (Leimar and Connor 2003), are also widespread (Bronstein et al. 2006). A by-product mutualism occurs when each of the species involved acts to optimise its own fitness, and the by-product of doing this is an increase in the fitness of the partner. Critically, there is no evolved investment in partner fitness, and these relationships tend to remain diffuse and non-specific (Leimar and Connor 2003). By-product mutualisms are expected to persist in novel ecosystems in which many native species are no longer present because the species involved are generalists and not reliant on a single partner. Hence, the interaction could persist under two scenarios: (1) a subset of the remaining partners continue to interact, or (2) invading non-natives fill the roles vacated by locally extinct partners.

Globally, many species with no recent shared evolutionary history are being brought together by the creation of novel ecosystems through anthropogenic habitat degradation and the transport of species around the world. Across the tropics, a major driver of such changes is selective logging of forests (Edwards et al. 2014b) and subsequent conversion to agriculture (Tilman et al. 2001). Selectively logged forest often retains similar numbers of species to primary forest across a range of taxa (Gibson et al. 2011), but has altered species composition (Edwards et al. 2014a). Conversion to agriculture typically has more extreme impacts (Edwards et al. 2014a; Gibson et al. 2011), with loss of forest species and invasion by non-natives (Mack et al. 2000)—and hence increased likelihood of the formation of novel species interactions. Although these non-native species are often considered in a negative context, they have the potential to be functionally important in novel ecosystems (Davis et al. 2011).

Mutualistic interactions between ants and plants are widespread, with plants trading housing or food for ant-provided protection from herbivores, seed dispersal or food collection (Bronstein et al. 2006). Mutualistic ant–plant networks are known to be fairly robust to habitat degradation (Bruna et al. 2005; Passmore et al. 2012), but the effects of degradation on the benefits for partners, at least for mutualisms where plants provide housing, are less well-known (Wetterer 1997). In the study reported here, we focussed on an ant–epiphyte interaction that persists across a disturbance gradient from primary forest to selectively logged forest and oil palm agriculture (Fayle et al. 2010; Turner and Foster 2009) in Malaysian Borneo, Southeast Asia. This region is a biodiversity hotspot (Myers 2000), which is suffering from an accelerating rate of deforestation (Hansen et al. 2013), driven mainly by expansion of oil palm plantations (Koh and Wilcove 2008). Bird’s nest ferns (*Asplenium* spp.) are litter-trapping epiphytes that form a two-way by-product mutualism in primary forest with their ant inhabitants, with ferns providing multiple colonies of ants with a nest site in their root mass and the ants protecting ferns from herbivores (Fayle et al. 2012). However, since the root mass is not enclosed or partitioned, being adapted for the capture and breakdown of leaf litter, ferns are unable to direct housing benefits to more beneficial ant partners. Larger ferns thus support more ant colonies of different species rather than larger colonies of cooperating species, which in turn means that ant species that are more beneficial are unlikely to benefit greatly from helping ferns to grow (i.e. they are less likely to receive partner fidelity feedbacks) (Fayle et al. 2012). This is in contrast to plants that house ant partners in adapted, enclosed structures (termed domatia) and are, consequently, able to direct benefits by rewarding or punishing specific ant colonies depending on their behaviour (Edwards et al. 2006).

Logging and conversion to oil palm plantation might affect the bird’s nest fern–ant interaction in a number of ways. Increasing homogenisation of ant faunas with disturbance, in particular compression of communities in response to vertical collapse of microclimate regimes (Fayle et al. 2009; Scheffers et al. 2013), is expected to reduce the specificity of the interaction (but see Beaulieu et al. 2010). Since bird’s nest ferns support substantial amounts of leaf litter, we would expect this collapse to result in increasing similarity between fern-dwelling ant communities and ant communities inhabiting leaf litter on the forest floor. Predation rates by ants are known to increase with disturbance (Tvardikova and Novotny 2012), and non-native species, which commonly invade disturbed areas, are often better protectors of plants than native species (Ness and Bronstein 2004). The relationship between fern size and the number and size of resident colonies has implications for the benefits to ants to invest in fern growth, and hence on the potential for partner fidelity feedbacks (Weyl et al. 2010). There are two possible disturbance-linked changes to these partner fidelity feedbacks: either resident ants expand colony sizes as fern size increases (and hence reap benefits from investment in fern fitness), or extra living space is co-opted by new ant colonies (and hence residents would not benefit from investment, as is the case in primary forest, see above). Non-native ant species, which can play key roles in mutualisms (Ness and Bronstein 2004), occur in bird’s nest ferns in oil palm plantations (Fayle et al. 2010), but their role in this by-product mutualism is not known. A species pool of functionally equivalent fern-dwelling ant species (both natives and non-natives), including those robust to habitat disturbance, would allow persistence of the by-product mutualism in this altered habitat.

We used the ant–fern model system to investigate whether a non-specific, two-way by-product mutualism can survive selective logging and conversion to oil palm plantation, with quantification of the relative roles of native and non-native species in driving this interaction in altered habitats. In this context, we tested three hypotheses: (1) that the degree of specificity of the ant–fern interaction, as measured by the overlap between fern-dwelling ants and the pool of potential colonists from leaf litter on the forest floor, will decrease with disturbance; (2) that the degree of protection from herbivory provided by the ants to their host ferns will increase with disturbance, since ant predation rates are known to be higher in more disturbed areas (Tvardikova and Novotny 2012); (3) that the potential for partner fidelity feedback between fern size and ant colony size will be unaffected by disturbance, since ant housing is not enclosed or partitioned in modified habitats, thereby limiting the degree of investment in fern growth by ants.

## Materials and methods

### Study sites

**Fig. 1:**
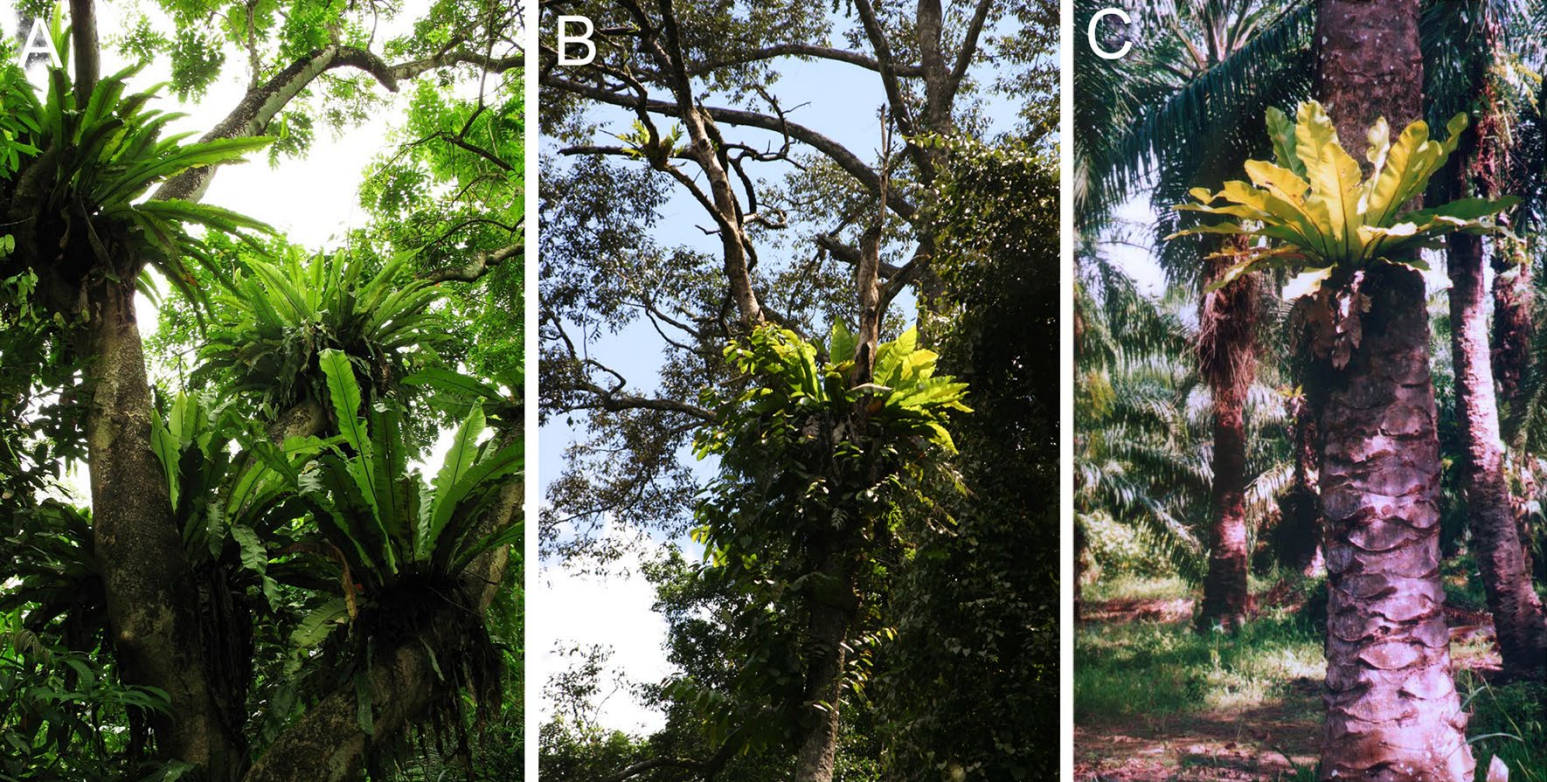
Bird’s nest ferns (*Asplenium* spp.) are abundant in habitats across a gradient of habitat modification in Southeast Asia, from primary forest (**a**) to logged forest (**b**) to oil palm plantation (**c**). Two closely related bird’s nest fern species co-exist in primary forest (their distribution is unknown in logged forest and oil palm plantation): *A. phyllitidis* and *A. nidus* (Fayle et al. 2009), with *A. phyllitidis* being by far the more abundant at the heights surveyed in this study. The two species are morphologically very similar and often only distinguishable using molecular characters

Bird’s nest ferns and ants were collected in May and June 2002 in three habitats in Sabah, Malaysian Borneo (Fig. 1): primary forest within the Danum Valley Conservation Area (117°48′E, 4°58′N, m a.s.l. 170 m), nearby forest that had been selectively logged once in 1988, with 113 m^3^/ha of timber removed (Fisher et al. 2011) (117°50′E, 4°57′N, m a.s.l. 170 m) and mature oil palm plantation (143 palms/ha), planted 14–18 years previously [118°35′E, 5°01′N, m a.s.l. 150 m; Electronic Supplementary Material (ESM) Fig. S1]. The climate of the area is aseasonal, although with occasional droughts (Walsh and Newbery 1999), none of which coincided with our sampling period.

### Ant–fern censuses

Twenty ferns were censused in each habitat (total *N* = 60) using a size-based random-stratified sampling scheme (frond tip to tip diameter: 0–50 cm, 6 ferns; 50–100 cm, 8 ferns; >100 cm, 6 ferns). This stratification was done to ensure maximal power to detect relationships between fern size, and ant community and colony responses in analyses of partner fidelity feedback. Each fern was collected into a plastic bag using ladders and the single rope technique, then weighed, dissected, and placed into a Winkler-type extractor for 3 days. Fern material was then searched by hand for remaining ants, dried to constant mass at 60 °C, and weighed again. Ants were identified to the genus level (Fayle et al. 2014) and then sorted to species/morphospecies using published keys (Agosti 1992; Bolton 1977; Kohout 2006; Shattuck 2008; Wang 2003) and the antweb.net online image database (Pfeiffer 2013). The presence of workers was used as an indication of the presence of a colony (191 occurrences of 87 species in total across all 3 habitats; ESM Table S1). Vouchers are deposited at the University Museum of Zoology, Cambridge, UK.

### Measuring mutualism specificity

Habitat modification could alter both the available ant partners and the proportions of those species residing in the ferns. We therefore surveyed leaf litter ant communities at the time of fern collection, collecting four 1-m^2^ litter samples as close to the focal tree as possible on bearings of 0°, 90°, 180° and 270°; these were processed in the same way as the fern samples. Compared with other microhabitats, ground-based leaf litter ant communities are likely to be most similar to the ant communities in the ferns, since both microhabitats comprise decomposing leaf litter. To assess overlap in ant communities between ferns and litter on the ground, we used the Chao–Jaccard index, which corrects for undersampling, with 200 bootstrapped replications to generate standard errors (Colwell 2009). We also assessed changes in the occupancy rates of ferns (binary factor) due to logging and conversion to oil palm plantation, using an ordered contingency table (*lbl_test* function in R package *coin;* Hothorn et al. 2008), on the assumption that changes would be monotonic.

### Assessing protection of ferns by ants

For each fern, we measured the total frond area and the area removed by herbivores and calculated the proportion of herbivory (following Fayle et al. 2012). We then used beta regression with a logit link function, a standard method for modelling proportions (Cribari-Neto and Zeileis 2010), to model the proportion of herbivory as a function of the number of ant colonies, total ant abundance (summed across all colonies in the fern), fern dry mass (since older, larger ferns were likely to have accumulated more herbivory), proportion of fern dry mass as leaf litter, fern core moisture content (grams moisture per gram dry mass fern) and habitat.

Ant exclusions were conducted in 2003 to verify any correlational patterns. On 20 additional ferns in each habitat (diameter 50–180 cm, height above ground level 0.8–3.3 m), we selected two fronds of similar size with minimal herbivory and assigned these to control and ant exclusion treatments at random. The ant exclusion frond base was covered with tanglefoot glue, while control fronds were left untreated. Any twigs or leaves in contact with the control or exclusion treatment fronds that would allow access by ants to the fronds were removed. Note that this approach is conservative in that non-flying herbivores were unable to access treatment (exclusion) fronds; hence our experiment assessed only the impacts of ant patrols on flying, ballooning, and jumping herbivores, as well as on any larvae developing from eggs laid by these species. After 2 months, the fronds were collected and the proportional area of herbivory measured and analysed using a generalised additive model (GAM) with beta error structure and a logit link (Stasinopoulos and Rigby 2007). We looked initially for an interaction between the effect of the experimental treatment and the effect of habitat, since this would indicate that ant protection of ferns was affected by habitat modification.

### Investigating partner fidelity feedbacks

We first analysed relationships between fern size and colony size for common ant species (those present in ≥5 ferns in each habitat), applying a sequential Bonferroni correction for multiple comparisons across species (Sokal and Rohlf 1995). We then analysed the overall relationship between fern size and total ant abundance, and between fern size and number of colonies. Linear models were used, and fern mass and ant abundance were log transformed for all analyses.

### Exploring the role of non-native ant species in modified habitats

Since non-native ant species are often widespread and ecologically important in modified habitats, we expected these to affect interaction specificity, ant protection and partner fidelity feedbacks. We defined non-natives as those categorised as invasive, alien or tramp by Pfeiffer et al. (2008). Excluding non-native species from our data, we repeated three of our analyses (final models only) assessing: (1) fern and litter community similarity; (2) herbivory rates and ant occupancy; (3) fern size and ant abundance/species richness. To test whether non-native species differed in their effects from the rest of the community, we used null models in which we ran each of these analyses a further 5,000 times with the same number of species (other than non-natives) removed at random. This methodology allowed us to test whether the observed analytical outputs were significantly different from what would be expected if non-native ant species were functionally indistinguishable from the rest of the ant community. *P* values were generated by comparing the observed output value to null distributions. These analyses were conducted only in oil palm plantation, since non-native species are not abundant elsewhere (primary forest 0/61 ant colonies; logged forest: 2/62 ant colonies). It should be noted that since a number of less well-known non-native species may not have been identified, our analyses represent a minimum (and conservative) estimate of the importance of non-native ants.

## Results

### Mutualism specificity and habitat modification

**Fig. 2:**
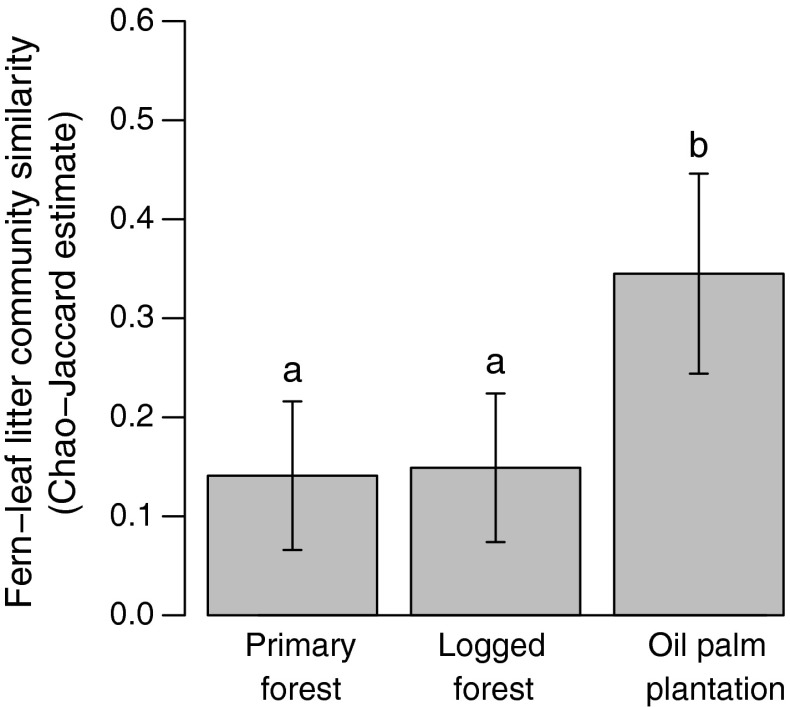
Similarity in species composition between fern-dwelling ant communities and those from ground leaf litter was greater in the oil palm habitat than in either the primary or logged forest, which did not differ from each other. *Bars* show mean incidence-based Chao–Jaccard similarities ± standard deviation (SD). *Different letters* denote significant differences between groups based on *t* tests on bootstrapped data (*N* = 200 replications)

Mutualism specificity was altered by habitat modification (Fig. 2). The degree of similarity between fern and litter communities was low in both primary and logged forest, and similarity did not differ significantly between these habitats (*t* test: *N* = 20, *t* = 0.3, *P* = 0.737; Fig. 2). However, community similarity between fern and litter ants was significantly higher in oil palm plantation [*t* tests: primary forest (primary)–oil palm plantation (oil palm): *N* = 20, *t* = 7.3, *P* < 0.001; logged forest (logged)–oil palm: *N* = 20, *t* = 7.0, *P* < 0.001]. The total number of ant species in the ferns was similar across the three habitats (primary: 36; logged: 35; oil palm: 35; ESM Table S1), while fern occupancy rates were reduced with increasing disturbance (primary: 100 %; logged: 85 %; oil palm: 75 %; ordinal contingency test: *χ* ^2^ = 5.32, *N* = 60, *P* = 0.021). There were relatively few fern-dwelling ant species in common between the habitats (primary–logged forest overlap 11 species; primary–oil palm overlap 2 species; logged–oil palm overlap 6 species). The reader is referred to Fayle et al. (2010) for further description of changes in ant community composition.

### Ant protection of ferns and habitat modification

**Fig. 3:**
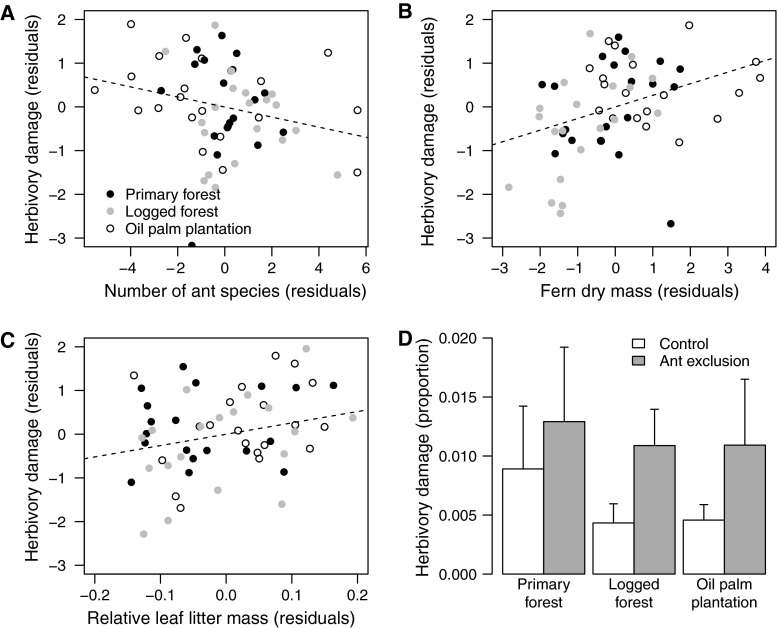
Herbivory rates were lower in ferns occupied by more colonies of ant (**a**), higher in larger ferns (**b**) and higher in ferns with greater accumulations of leaf litter (**c**). These effects did not differ between habitats. Experimental exclusion of ants resulted in increased rates of herbivory across primary forest, logged forest and oil palm plantation (**d**), with no differences between habitats in this effect. Note that treatments and controls are paired. Mean proportions of frond removed by herbivores after 2 months are plotted with standard errors (SE). The regression plots (**a**–**c**) are generated using partial regression analyses, in which the *x-axis* values are the residuals from a regression of all other independent variables against the independent variable of interest, and the *y-axis* values are the residuals of a regression predicting herbivory rates using all independent variables other than the one of interest

Across all habitats, ferns with more colonies of ants experienced lower levels of herbivory (beta regression: *Z*_5,58_ = −2.07, *P* = 0.039; Fig. 3a), while larger ferns (*Z*_5,58_ = 2.82, *P* = 0.005; Fig. 3b) and those with more leaf litter (*Z*_5,58_ = 2.04, *P* = 0.042; Fig. 3c) experienced higher levels of herbivory. There was no effect of habitat, fern core moisture content or ant abundance on herbivory (*P* > 0.2). For a fern of average size (dry mass 249.0 g) and with an average proportion of its dry mass as leaf litter (0.167), the predicted leaf area missing through herbivory is 11.9 % for a fern occupied by six ant colonies, increasing to 17.2 % for an uninhabited fern (45 % difference). We found that experimental ant exclusion increased herbivory across all habitats (GAM: *t*_43,74_ = 2.59, *P* = 0.011; Fig. 3d), with no differences between habitats in terms of the strength of this effect (primary vs. oil palm: *t*_43,74_ = 1.60, *P* = 0.119; logged vs. oil palm: *t*_43,74_ = 0.37, *P* = 0.710, primary vs. logged: *t*_43,74_ = 1.19, *P* = 0.236) and no significant interaction between habitat type and effect of ant exclusion (all *P* > 0.05). Ant exclusion increased the leaf area lost to herbivores over a 2-month period from 0.6 to 1.2 % (95 % increase across all habitats).

### Partner fidelity feedback and habitat modification

**Fig. 4:**
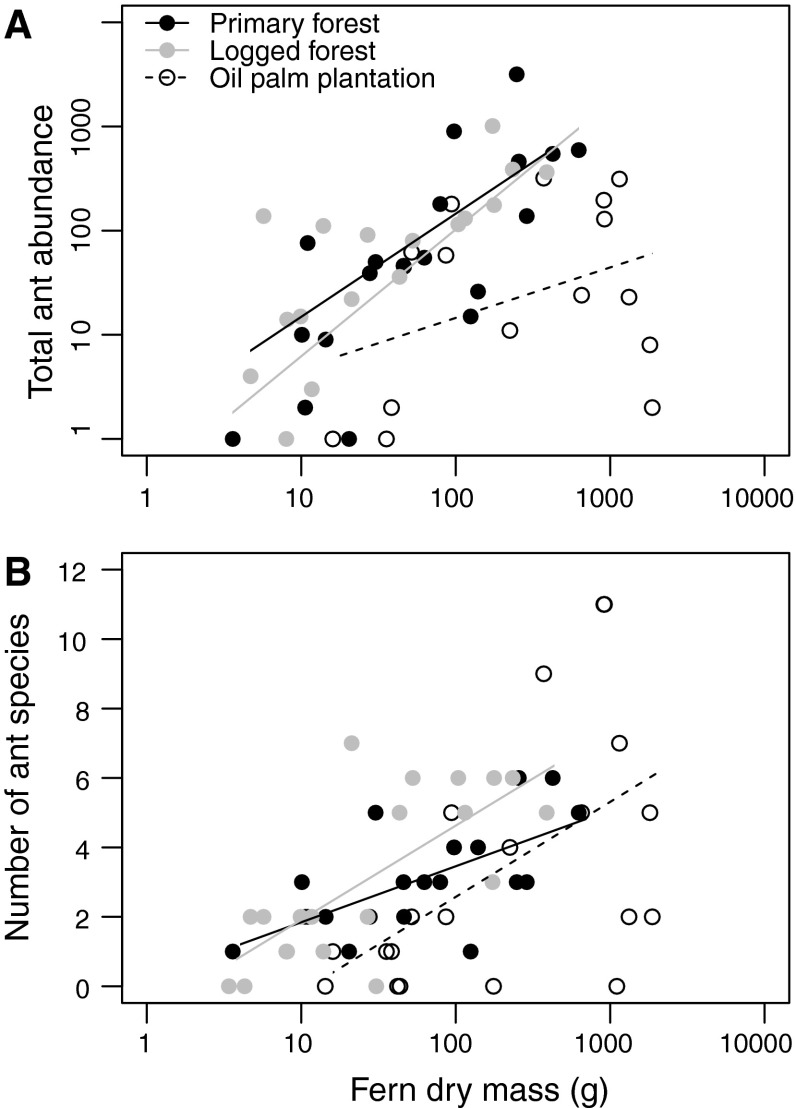
Total ant abundance (**a**) and the number of ant species present (**b**) were greater in larger ferns. For a given size, ferns in oil palm plantation supported lower abundances of ants than ferns in primary or logged forest, and fewer ant species than ferns in logged forest

For common ant species (≥5 colonies in a habitat), there was no relationship between colony size and fern size, although larger ferns supported higher total abundances of ants (Fig. 4a). However, this relationship was habitat dependent (Linear model; fern size × habitat interaction: *t*_5,54_ = −2.36, *P* = 0.022), with abundance increasing more slowly with fern size in the oil palm plantation than in forest habitats (Fig. 4a). Larger ferns were also inhabited by more species of ant (Linear model: *t*_3,56_ = 5.60, *P* < 0.001; Fig. 4b), and ferns in primary forest did not differ from those in logged forest or oil palm plantation in this relationship. In contrast, ferns in logged forest supported more ant species than ferns in oil palm plantation for a given fern size (primary vs. logged: *t*_3,56_ = 1.15, *P* = 0.254, primary vs. oil palm: *t*_3,56_ = 1.36, *P* = 0.179; logged vs. oil palm: *t*_3,56_ = 2.30, *P* = 0.026). There were no significant first-order interactions between any of the predictors in analyses of species richness.

### The role of non-native ant species in mutualism in oil palm plantation

**Fig. 5:**
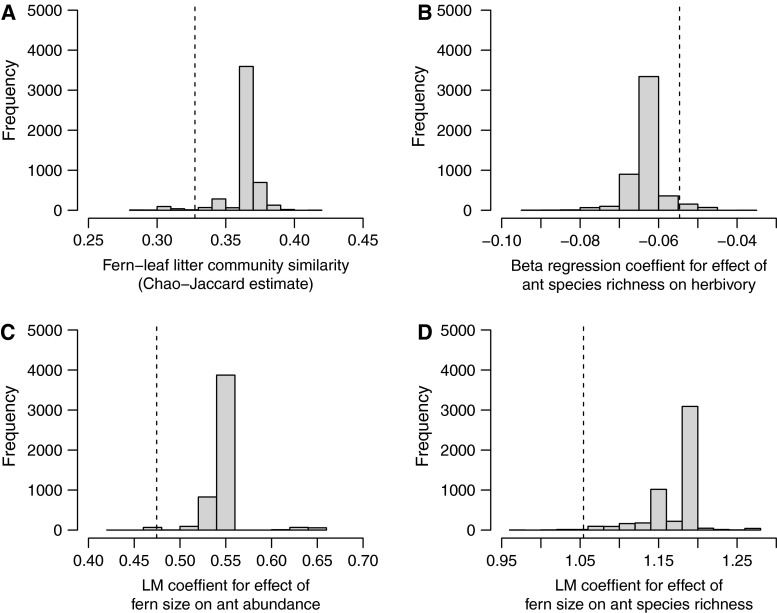
Exclusion of non-native species from analyses in the oil palm plantation resulted in no difference in similarity between fern and leaf litter ant communities (**a**), no difference in the correlation between ant species richness and herbivory rates (**b**), a weaker relationship between ant abundance and fern size (**c**), and a weaker relationship between ant species richness and fern size (**d**). *Vertical lines* denote single parameter values derived from removal of all non-native species, *histograms* plot distributions of parameter values with the same number of randomly selected other species repeatedly removed. *LM* stands for Linear Model

There was no difference in the degree of similarity between fern-dwelling and litter-dwelling ant communities, or in the relationship between ant species richness and herbivory, when analyses excluding non-native species from the oil palm habitat dataset were carried out (Chao–Jaccard metric: non-natives removed 0.328, null median 0.364, *P* = 0.061; Fig. 5a; beta regression coefficient: non-natives removed −0.055, null median −0.062, *P* = 0.089; Fig. 5b). However, the relationship between fern size and ant abundance (Linear model coefficient for fern size: non-natives removed 0.474, null median 0.542, *P* = 0.006; Fig. 5c) and that between fern size and number of colonies (Linear model coefficient for fern size: non-natives removed 1.05, null median 1.17, *P* = 0.010, two tailed test; Fig. 5d) was weaker with non-native species removed than with them present, indicating that non-native species are more important than the rest of the community in contributing to these relationships.

## Discussion

The ongoing anthropogenic modification of global ecosystems is bringing together novel combinations of species (Hobbs et al. 2009). This is expected to cause shifts in the functioning of ecosystems, with associated changes to the costs or benefits of specific interactions. For ants engaged in a by-product mutualism with bird’s nest ferns, we found that the interaction persisted following disturbance by selective logging and conversion to oil palm plantation and that ant protection of ferns is maintained, despite a reduction in the mutualism specificity and lower occupancy rates in oil palm ferns. This contrasts with the negative impacts of anthropogenic disturbance observed for a range of other mutualisms (Kiers et al. 2010; Potts et al. 2010).

The persistence of the ant–fern interaction probably relates to the low degree of specificity between ants and bird’s nest ferns. In primary forest, at least 71 species of ants inhabit the ferns (survey of 83 ferns; Fayle et al. 2012). Because fern cores are not enclosed or partitioned, they are unable to filter ant partners (e.g. by constraining the shape of a preformed entrance hole; Brouat et al. 2001) or to direct punishments (e.g. by aborting structures supporting cheating colonies; Edwards et al. 2006) as occurs in domatia-provisioning ant–plant mutualisms (those in which plants provide enclosed structures adapted for ant inhabitation). Hence, the non-specific nature of the ant–bird’s nest fern by-product mutualism appears to allow the persistence of this relationship even in a novel ecosystem. Indeed, the relationship becomes even less specific in oil palm plantation (Fig. 2), indicating a homogenisation of the fern-dwelling and litter-dwelling ant faunas. We therefore predict that in novel ecosystems generated by significant disturbance, highly specific interactions will be more likely to go extinct (Koh et al. 2004), with only a core of non-specific interactions (such as by-product mutualisms) remaining. This “skeletonisation” of interaction networks is likely to have consequences for the way that novel ecosystems function and could prove a fruitful direction for further research.

One consequence of the persistence of ant–fern interactions that we noted was the continued protection of ferns from herbivory in disturbed habitats (Fig. 1a, d). Such robustness is presumably driven by foraging of resident ants on vegetation near to their nest for insect prey, with the effect persisting despite lower overall abundances of ants in oil palm plantations (Fig. 4a). One possible explanation is that lower ant abundances are compensated for by increased levels of patrolling, due either to higher temperatures in this habitat (Turner and Foster 2006) or by a combination of species turnover and species-specific differences in patrolling rates (Bruna et al. 2004). Larger ferns exhibited more herbivory, probably because they were older and, consequently, their fronds had been exposed to herbivores for a longer period of time. Ferns with more leaf litter also experienced more herbivory, possibly because a larger volume of leaf litter is likely to buffer microclimate more efficiently (Turner and Foster 2006) and hence act as a refuge for herbivores. The difference in magnitude between observed levels of herbivore damage (11.9 % for a fern of average size and litter mass) and those found on control ferns (0.6 % over a 2-month period) indicates that fronds may be long-lived and, hence, that there may be high costs of damage, particularly early in development. The persistence of ant protection of ferns is likely to reflect the continuing control of herbivores in the wider oil palm landscape (Wielgoss et al. 2014). Therefore, species known to be involved in non-specific interactions in pristine habitats may be the most promising candidates for delivering services in degraded and novel ecosystems. However, in our study, the increased overlap between ground-dwelling and fern-dwelling ant communities indicates that the by-product mutualism in oil palm is dependent on a larger proportion of species present in the habitat as a whole. This might lead to increased vulnerability of fern protection to ant species extinction.

The overall structure of partner-fidelity feedbacks remained the same in the disturbed habitats, with larger ferns generally supporting more colonies of ants, rather than larger colonies of particular ant species. However, oil palm ferns supported lower overall abundances of ants for a given size (and also fewer colonies) than in logged forest ferns. Furthermore, the proportion of occupied ferns decreased with increasing disturbance. Lower ant occupation of oil palm ferns could be due to a reduction in the volume of ferns suitable for habitation: due to the hotter, drier microclimate in plantations (Foster et al. 2011; Turner and Foster 2006) the root mass dries out more rapidly, potentially leaving only the centre of the fern habitable [fern moisture content: 0.85 g water/g dry mass in oil palm ferns vs. 1.53 g/g in primary and 1.42 g/g in logged forest ferms; Fig. ESM S2; see Scheffers et al. (2014) for this effect on frogs]. Despite the lower ant abundance and species richness in oil palm plantation, there was no increase in the proportion of ferns supporting single ant colonies, which would be expected to lead to closer relationships between fern size and ant colony size, and hence for an increase in the potential for partner fidelity feedback creation (primary forest: 4/20 ferns, logged forest: 3/20, oil palm: 3/20).

The persistence of by-product mutualism in the highly disturbed oil palm plantation habitat was reliant to some extent on the presence of non-native species. On a per-species basis, these non-native species were more important than the rest of the community in terms of the relationships between fern size and ant abundance, and between fern size and species richness. Non-native species were not significantly different from the rest of the community in terms of their predicted contribution to fern protection. Widespread invasive and tramp species are well documented as disruptors of animal–plant mutualisms (Traveset and Richardson 2006). Conversely, these species are often locally highly abundant (Mack et al. 2000), and hence their potential to function in by-product or generalist mutualisms may be even greater than that of native species (Aizen et al. 2008). Indeed, in oil palm plantations, non-native species are widespread both on the ground and in the canopy (Fayle et al. 2010), with the canopy sometimes supporting even higher densities of non-natives than are found in the ferns (18 % of occurrences in ferns in this study; 46 % of occurrences in the canopy in Pfeiffer et al. 2008).

The high abundance of non-native ants elsewhere in the environment, combined with the non-specific nature of the by-product mutualism, seems to have allowed non-native ants to take the place of some of the rain forest species that were lost when habitats were modified. There could, therefore, be a shift in the selective pressures on the partners involved in this relationship, with a potential for evolutionary changes over longer time periods. However, since the mutualism is a by-product of otherwise adaptive behaviours for both partners, there is probably less potential for such changes than there would be for a more intimate novel relationship. Invasion by non-natives also potentially changes the adaptive landscape for remaining native ant species, particularly if non-natives are highly competitive, although this appears not to be the case for ants inhabiting oil palm ferns (Fayle et al. 2013). It should be noted that, in our study, non-native species in oil palm ferns comprised only a minority of the overall species occurrences (18 %), and of the non-native occurrences we observed in ferns, 50 % were of a single species, *Monomorium floricola*. Hence, the broader impact of non-natives in this system remains unclear.

In conclusion, we have demonstrated that a by-product mutualism persists along a gradient of habitat modification, with no detectable changes in the benefits for the partners. We also found that non-native species may play roles in such generalist mutualisms in highly disturbed habitats. Our results support the notion that management practices should take into account these robust interactions and functions in novel ecosystems and tailor management efforts to support them (Hobbs et al. 2009; Melo et al. 2013). As the footprint of human disturbance continues to expand globally, so the role of novel species and ecosystems will increase. The consequences of this expansion on the linkages that bind together species into communities and the effects of these changes on species’ evolutionary trajectories are only just starting to be understood.

## Electronic supplementary material

Below is the link to the electronic supplementary material.

Supplementary material (DOCX 1947 kb)
