# Supplementary material for: An ant–plant by-product mutualism is robust to selective logging of rain forest and conversion to oil palm plantation
Source: Oecologia. 2015 Jan 10;178(2):441–50. doi: 10.1007/s00442-014-3208-z (PMC4439435; doi:10.1007/s00442-014-3208-z)
Supplement: Supplementary file 1 — Supplementary material (DOCX 1947 kb) [file 442_2014_3208_MOESM1_ESM.docx]

**An ant-plant by-product mutualism is robust to selective logging of rain forest and conversion to oil palm plantation**

Tom M. Fayle* ^a,b^, David P. Edwards ^c, d^, William A. Foster ^e^, Kalsum Mohd Yusah ^b,f^, Edgar C. Turner ^e^
^a^ Faculty of Science, University of South Bohemia and Institute of Entomology, Biology Centre of the Academy of Sciences Czech Republic, Branišovská 31, 370 05 České Budějovice, Czech Republic

^b^ Forest Ecology and Conservation Group, Imperial College London, Silwood Park Campus, Buckhurst Road, Ascot, Berkshire, SL5 7PY, UK

^c^ Department of Animal and Plant Sciences, University of Sheffield, Western Bank, Sheffield, S10 2TN, UK

^d^ Centre for Tropical Environmental and Sustainability Science (TESS) and School of Marine and Tropical Biology, James Cook University, Cairns, Queensland, Australia

^e^ Insect Ecology Group, University Museum of Zoology Cambridge, Downing Street, Cambridge CB2 3EJ, UK

^f^ Institute for Tropical Biology and Conservation, Universiti Malaysia Sabah, Jalan UMS, 88400 Kota Kinabalu, Sabah, Malaysia

*Corresponding author: tmfayle@gmail.com


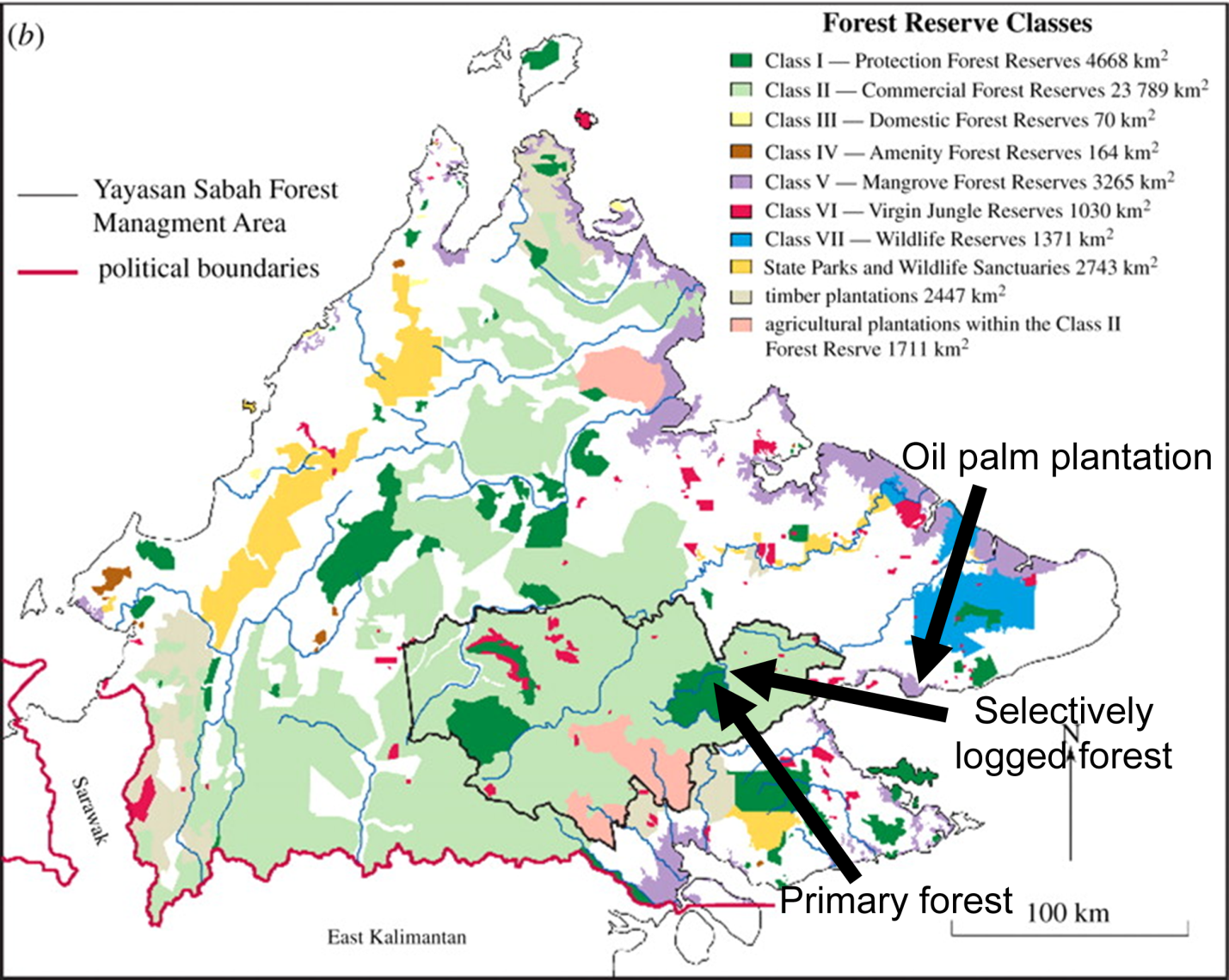


Figure S1. The locations of the three habitats within Sabah, Malaysian Borneo in which surveys and experimental manipulations were conducted. Reproduced, with permission, from Reynolds et al. (2011). *Phil. Trans. Roy. Soc. B.* 366: 3168-3176.

Figure S2. Fern core moisture content varied with habitat conversion (Linear model: F_2,57_=3.90, P=0.026), being higher in both primary and logged forest than in oil palm (primary vs. oil palm: t_2,57_=2.60, P=0.012; logged vs. oil palm: t_2,57_=2.18, P=0.033). Moisture content of ferns in primary and logged forest did not differ (t_2,57_=0.42, P=0.678). The final model included habitat, but not fern mass or an interaction between fern mass and habitat. Different letters denote significantly different means (P<0.05).

Table S1. Species found to inhabit bird’s nest ferns in primary forest, logged forest and oil palm plantation. Numbers are counts of colonies observed in twenty ferns (N=20 per habitat). Morphospecies numbers are given where species names were not assigned. *Non-native species, designated as being invasive, alien or tramp following [Pfeiffer et al. (2008](#_ENREF_24)).

| Subfamily | Genus | Species | Primary forest | Logged forest | Oil palm plantation |
| --- | --- | --- | --- | --- | --- |
| Amblyoponinae | *Prionopelta* | *kraepelini* | 0 | 0 | 3 |
| Dolichoderinae | *Dolichoderus* | nr. *cuspidatus* | 2 | 0 | 0 |
| Dolichoderinae | *Dolichoderus* | Y | 1 | 1 | 0 |
| Dolichoderinae | *Tapinoma* | B1 | 0 | 0 | 2 |
| Dolichoderinae | *Tapinoma* | T89 | 0 | 0 | 1 |
| Dolichoderinae | *Technomyrmex* | T104 | 0 | 1 | 0 |
| Dolichoderinae | *Technomyrmex* | T116 | 2 | 0 | 0 |
| Ectatomminae | *Gnamptogenys* | nr. *treta* | 2 | 0 | 0 |
| Formicinae | *Anoplolepis* | *gracilipes** | 0 | 0 | 1 |
| Formicinae | *Camponotus* | ?*arrogans* | 0 | 1 | 0 |
| Formicinae | *Camponotus* | T1 | 1 | 3 | 0 |
| Formicinae | *Camponotus* | XZ5 | 0 | 2 | 0 |
| Formicinae | *Myrmoteras* | *marianneae* | 0 | 1 | 0 |
| Formicinae | *Paratrechina* | A | 0 | 3 | 0 |
| Formicinae | *Paratrechina* | FR44 | 3 | 1 | 0 |
| Formicinae | *Paratrechina* | *longicornis** | 0 | 0 | 2 |
| Formicinae | *Paratrechina* | RF39 | 1 | 0 | 0 |
| Formicinae | *Paratrechina* | T101 | 0 | 2 | 0 |
| Formicinae | *Paratrechina* | T31 | 2 | 0 | 0 |
| Formicinae | *Plagiolepis* | Y1 | 0 | 3 | 6 |
| Formicinae | *Polyrhachis* | T38 | 2 | 0 | 0 |
| Formicinae | *Polyrhachis* | *danum* | 1 | 0 | 0 |
| Formicinae | *Polyrhachis* | nr. *armata* | 0 | 1 | 0 |
| Formicinae | *Polyrhachis* | nr. *mitrata* | 1 | 0 | 0 |
| Formicinae | *Polyrhachis* | *proxima* | 1 | 0 | 0 |
| Formicinae | *Pseudolasius* | T22 | 0 | 0 | 1 |
| Myrmicinae | *Carebara* | B84 | 1 | 0 | 0 |
| Myrmicinae | *Carebara* | DH7 | 0 | 0 | 1 |
| Myrmicinae | *Carebara* | T233 | 0 | 1 | 0 |
| Myrmicinae | *Carebara* | T32 | 2 | 0 | 4 |
| Myrmicinae | *Carebara* | T97 | 1 | 1 | 0 |
| Myrmicinae | *Crematogaster* | B108 | 1 | 2 | 0 |
| Myrmicinae | *Crematogaster* | nr. *modiglianii* | 0 | 1 | 0 |
| Myrmicinae | *Crematogaster* | Q1 | 0 | 1 | 0 |
| Myrmicinae | *Crematogaster* | *rogenhoferi* | 0 | 1 | 0 |
| Myrmicinae | *Crematogaster* | T108 | 1 | 0 | 0 |
| Myrmicinae | *Crematogaster* | T109 | 2 | 0 | 0 |
| Myrmicinae | *Crematogaster* | T110 | 3 | 0 | 0 |
| Myrmicinae | *Crematogaster* | T95 | 0 | 1 | 2 |
| Myrmicinae | *Lophomyrmex* | B14 | 0 | 0 | 1 |
| Myrmicinae | *Monomorium* | B44 | 0 | 2 | 2 |
| Myrmicinae | *Monomorium* | C1 | 0 | 0 | 1 |
| Myrmicinae | *Monomorium* | *floricola** | 0 | 2 | 6 |
| Myrmicinae | *Monomorium* | RF22 | 0 | 1 | 3 |
| Myrmicinae | *Monomorium* | T90a | 0 | 0 | 1 |
| Myrmicinae | *Monomorium* | T98 | 3 | 5 | 0 |
| Myrmicinae | *Pachycondyla* | *obscurans* | 0 | 0 | 1 |
| Myrmicinae | *Pheidole* | ?*sauberi* | 0 | 2 | 0 |
| Myrmicinae | *Pheidole* | B6 | 0 | 4 | 0 |
| Myrmicinae | *Pheidole* | B63 | 0 | 1 | 0 |
| Myrmicinae | *Pheidole* | B99 | 0 | 0 | 1 |
| Myrmicinae | *Pheidole* | RF19 | 3 | 0 | 0 |
| Myrmicinae | *Pheidole* | RF50 | 0 | 1 | 0 |
| Myrmicinae | *Pheidole* | T107 | 0 | 2 | 0 |
| Myrmicinae | *Pheidole* | T80 | 0 | 0 | 2 |
| Myrmicinae | *Pheidole* | T83 | 0 | 0 | 1 |
| Myrmicinae | *Pheidologeton* | T85 | 0 | 0 | 1 |
| Myrmicinae | *Pristomyrmex* | *brevispinosus* | 1 | 0 | 0 |
| Myrmicinae | *Pyramica* | T99 | 1 | 1 | 0 |
| Myrmicinae | *Solenopsis* | B43 | 1 | 0 | 3 |
| Myrmicinae | *Strumigenys* | T78 | 0 | 0 | 4 |
| Myrmicinae | *Strumigenys* | T86 | 0 | 0 | 1 |
| Myrmicinae | *Strumigenys* | T93 | 0 | 0 | 1 |
| Myrmicinae | *Tetramorium* | *eleates* | 0 | 0 | 1 |
| Myrmicinae | *Tetramorium* | *noratum* | 0 | 1 | 0 |
| Myrmicinae | *Tetramorium* | nr. *carinatum* | 0 | 0 | 1 |
| Myrmicinae | *Tetramorium* | *pacificum** | 0 | 0 | 1 |
| Myrmicinae | *Tetramorium* | T111 | 1 | 0 | 0 |
| Myrmicinae | *Tetramorium* | T118 | 1 | 0 | 0 |
| Myrmicinae | *Tetramorium* | T81 | 0 | 1 | 4 |
| Myrmicinae | *Tetramorium* | *tonganum** | 0 | 0 | 2 |
| Myrmicinae | *Vollenhovia* | T113 | 1 | 0 | 0 |
| Myrmicinae | *Vollenhovia* | WW | 0 | 0 | 2 |
| Ponerinae | *Cryptopone* | T211 | 0 | 0 | 1 |
| Ponerinae | *Diacamma* | RF36 | 8 | 6 | 0 |
| Ponerinae | *Diacamma* | *rugosum* | 0 | 0 | 2 |
| Ponerinae | *Diacamma* | T114 | 2 | 0 | 0 |
| Ponerinae | *Hyponera* | T192 | 1 | 0 | 0 |
| Ponerinae | *Hypoponera* | JJ | 1 | 3 | 0 |
| Ponerinae | *Hypoponera* | T112 | 2 | 0 | 0 |
| Ponerinae | *Hypoponera* | T84 | 0 | 0 | 1 |
| Ponerinae | *Leptogenys* | T115 | 1 | 0 | 0 |
| Ponerinae | *Pachycondyla* | *tridentata* | 1 | 1 | 0 |
| Ponerinae | *Ponera* | DH1 | 0 | 1 | 0 |
| Ponerinae | *Ponera* | T105 | 2 | 1 | 0 |
| Ponerinae | *Ponera* | T119 | 1 | 0 | 0 |
| Proceratiinae | *Discothyrea* | T91 | 0 | 0 | 1 |
| **Total occurrences:** | | | 61 | 62 | 68 |
| **Total species richness:** | | | 36 | 35 | 35 |
